# Evaluation of antioxidant activities of extracts from 19 Chinese edible flowers

**DOI:** 10.1186/2193-1801-3-315

**Published:** 2014-06-25

**Authors:** Youwei Zeng, Maocheng Deng, Zhencheng Lv, Yonghong Peng

**Affiliations:** Department of Food and Bio-engineering, Guangdong Light Industry Technical College, Guangzhou, 510300 Guangdong China; Centre of Guangdong Higher Education of Engineering and Technological Development of Speciality Condiments, Guangdong Light Industry Technical College, Guangzhou, 510300 Guangdong China; Department of Life Science, Huizhou University, Huizhou, 516007 Guangdong China; College of Life Science, Guangdong Key Lab of Biotechnology for Plant Development, South China Normal University, Guangzhou, 510631 Guangdong China

**Keywords:** Edible flowers, Extracts, Free radical scavenging activity, Polyphenolic content, Flavonoid content

## Abstract

Extracts of 19 selected edible flowers were investigated for their free radical scavenging activity (FRSA), polyphenolic contents and flavonoid contents in the paper. The results showed the extracts of *Paeonia suffruticosa* Andr., *Paeonia lactiflora* Pall., and *Rosa rugosa* Thunb. possessed obviously stronger DPPH FRSA (94.221 ± 0.102; 93.739 ± 0.424 and 94.244 ± 0.163%, respectively), superoxide FRSA (55.818 ± 1.518; 52.142 ± 1.374 and 57.321 ± 0.608%, respectively), hydroxyl FRSA (85.872 ± 0.873; 89.307 ± 0.803 and 88.560 ± 0.277%, respectively) and polyphenolic contents (96.208 ± 0.689; 87.938 ± 1.187 and 92.164 ± 0.799 mg CE/g, respectively) that were superior or comparable to black and green teas. Polyphenolic contents did correlate well with DPPH FRSA (*r* = 0.943, *P* < 0.01), superoxide FRSA (*r* = 0.833, *P* < 0.01), and hydroxyl FRSA (*r* = 0.500, *P* < 0.05). It indicated that this potent FRSA may be attributed to its phenolic compounds. These findings showed that the tested flowers could be considered as new sources of safe natural antioxidants and preservatives of food industry.

## Introduction

Roles of the reactive oxygen species (ROS) and free radicals such as superoxide anion radicals, hydrogen peroxide and hydroxyl radicals are increasingly recognized in physiological process, and pathogenesis of many diseases (Moskovitz et al. 2002; Balaban et al. 2005). Their action is opposed by a balanced system of antioxidant defense, and excessive amount of ROS can initiate toxic and lethal chain reactions, which leads to cell damage and health problems (Aruoma 1998; Saeidnia and Abdollahi 2013). Recently, there is a growing interest in substances from natural sources exhibiting antioxidant properties that can be used to protect human beings from oxidative stress damage (Kris-Etherton et al. 2002). Substantive experiments have been focused on the phytochemicals and extracts from plants sources possessing antioxidant effects. Reports indicate that there is an inverse relationship between the dietary intake of antioxidant-rich plant source foods and the incidence of human disease (Sies 1993). However, many synthetic antioxidants such as butylated hydroxytoluene (BHT) and butylated hydroxyanisole (BHA) are widely used as food additives and may be responsible for liver damage and carcinogenesis (Williams et al. 1999).

Therefore, the development and utilization of more effective and non-toxic antioxidants from natural products are desired, not only for the food and drug storage, but also for the nutritional and clinical applications. A great deal of effort has focused on using available experimental techniques to identify natural antioxidants from medicinal plants (André et al. 2010). It is well known that the traditional Chinese herbs have been used in food and medicine over two thousand years (Wang et al. 2012). Those herbs may contain a wide variety of chemical composition, including phenolic compounds (e.g. phenolic acids, flavonoids, quinones, coumarins, lignans, stilbenes, tannins), nitrogen compounds (including alkaloids), vitamins, terpenoids (including carotenoids), with potential antioxidant activities (Cai et al. 2006). It showed herbs possessing anti-inflammatory, antiatherosclerotic, hypolipidemic, antiplatelet, antitumor, or immune-stimulating properties might be proper candidates to help reduce the risk of cardiovascular disease and cancer (Krishnaiah et al. 2011).

Edible herbal resources can provide enormous scope in correcting the imbalance through regular intake of proper diet. The objectives of this study is to survey the free radical scavenging activity (FRSA), total phenolic contents and total flavonoid contents of 19 selected species that are very popular as herbal flower teas in China, with comparisons with black and green teas of *Camellia sinensis* carried out as positive controls, in view of their potential benefits of natural antioxidants for food and medicinal purposes.

## Materials and methods

### Materials

Herbal flowers, together with black and green teas of *C. sinensis*, screened for FRSA are listed in Table 1. The dried herbal flowers were purchased form Yuyi Co., Ltd. Shanghai, black and green teas were Lipton products Unilever, and their origins were identified and proved by the College of Life Science South China Normal University. The flowers were dried, and then ground into fine particles with a special grinder for food processing. Sample (2.00 g) was suspended and extracted by refluxing with 50 ml boiling distilled water for 30 min. After cooling the extracts were filtered through a filter paper and the filtrates were freeze-dried. Analyses of aqueous extracts were done in triplicate. The dried extracts were diluted to a contraction of 1 mg/ml and stored at 4°C for further analysis.

**Table 1:** **Free radical scavenging activities of various herbal flowers and their polyphenolic contents and flavonoid contents** ^**a**^

Plant names	FRSA (%)			PC (mg CE/g)	FC (mg CE/g)
	DPPH ^•^	^.^OH	O		
*Chrysanthemum indicum* L.	14.125 ± 0.313	54.450 ± 1.539	18.538 ± 0.413	13.043 ± 0.315	44.629 ± 0.921
*Chrysanthemum morifolium* Ramat	11.970 ± 0.296	33.513 ± 0.844	0.431 ± 0.116	5.974 ± 0.148	10.837 ± 0.336
*Dianthus carryophylus* L.	13.528 ± 0.651	43.488 ± 1.610	5.058 ± 1.152	6.835 ± 0.449	53.248 ± 0.091
*Hibiscus sabdariffa* Linn.	18.916 ± 0.732	93.220 ± 0.346	21.723 ± 1.325	8.296 ± 0.293	24.469 ± 0.444
*Jasminum sambac* (L.) Ait.	13.047 ± 0.576	78.375 ± 0.656	0.405 ± 0.268	10.174 ± 0.230	54.357 ± 0.319
*Lavandula angustifolia* Mill.	47.213 ± 0.373	67.055 ± 1.685	40.290 ± 2.033	20.426 ± 0.499	27.392 ± 1.421
*Lilium longiflorum* Thumb.	13.987 ± 0.363	84.737 ± 0.235	15.124 ± 0.774	1.070 ± 0.148	22.123 ± 0.168
*Lonicera japonica* Thunb.	50.789 ± 1.307	60.842 ± 1.584	42.277 ± 0.704	32.113 ± 1.126	34.112 ± 0.543
*Matthiola incana* (L.) R. Br.	19.284 ± 0.304	78.973 ± 1.438	1.802 ± 0.989	7.069 ± 0.148	36.672 ± 0.277
*Osmanthus fragrans* (Thunb.) Lour.	54.963 ± 0.596	52.210 ± 0.695	58.420 ± 0.842	47.452 ± 1.855	20.373 ± 0.241
*Paeonia lactiflora* Pall.	93.739 ± 0.424	85.872 ± 0.873	55.818 ± 1.518	87.938 ± 1.187	28.757 ± 0.419
*Paeonia suffruticosa* Andr.	94.221 ± 0.102	89.307 ± 0.803	52.142 ± 1.374	96.208 ± 0.689	38.933 ± 0.770
*Panax ginseng* C. A. Mey	9.791 ± 0.098	58.124 ± 1.453	5.624 ± 1.990	6.652 ± 0.169	16.853 ± 0.348
*Panax notoginseng* (Burk.) F. H. Chen	8.119 ± 0.564	66.308 ± 1.136	7.724 ± 1.940	1.200 ± 0.037	11.115 ± 0.109
*Papaver rhoeas* L.	17.037 ± 0.155	39.337 ± 1.248	14.824 ± 1.272	4.878 ± 0.369	47.680 ± 1.029
*Prunus persica* (L.) Batsch	35.999 ± 0.827	60.335 ± 1.262	27.926 ± 1.736	20.713 ± 0.718	60.139 ± 0.884
*Rosa rugosa* Thunb.	94.244 ± 0.163	88.560 ± 0.277	57.321 ± 0.608	92.164 ± 0.799	77.312 ± 0.732
*Tagetes erecta* L.	17.150 ± 0.813	48.059 ± 0.680	19.847 ± 1.246	14.139 ± 0.369	42.453 ± 0.845
*Trollius chinensis* Bunge	66.152 ± 0.952	78.554 ± 1.176	38.884 ± 0.376	18.626 ± 0.293	83.797 ± 0.884
Black tea P.E. *Camellia sinensis*	85.322 ± 1.019	92.204 ± 0.253	48.139 ± 0.534	60.704 ± 1.233	60.288 ± 1.694
Green tea P.E. *Camellia sinensis*	93.191 ± 0.815	78.047 ± 1.847	59.169 ± 1.571	88.356 ± 1.489	26.667 ± 0.732

^a^FRSA = Free radical scavenging activity, PC = Polyphenolic content, FC = Flavonoid content. The mean values were obtained from triplicate experiments, the concentration of extracts were 1 mg/ml.

### DPPH free radical scavenging assay

Effects of extracts on DPPH^•^ based on the method modified by Sharma and Bhat (2009). 0.1 ml of aqueous extract was added to 2.4 ml of 0.12 mM DPPH^•^ solution and the mixture was shaken vigorously, after incubation at 25°C for 30 min, the absorbance was read at 517 nm against a blank of 50% ethanol using a Shimadzu UV-1206 ultraviolet–visible spectrophotometer (Shimadzu, Japan). The free radical scavenging activity (FRSA) was calculated using the following equation:


Where A_blank_ is the absorbance of the control reaction (containing all of the reagents except the test extract) and A_sample_ is the absorbance of the test samples. Each assay was performed in triplicate.

### Hydroxyl radical scavenging assay

The hydroxyl FRSA was assayed by using the 1,10-phenanthroline-Fe^2+^ oxidative method (Jin et al. 1996). The reaction mixture contained 0.15 ml of 5 mM 2-deoxyribose, 0.4 ml of 0.75 M sodium phosphate buffer solution (PBS, pH 7.4), 0.25 ml of H_2_O, 0.1 ml of 7.5 mM FeSO_4_, 0.1 ml of 1% H_2_O_2_ and 0.1 ml of sample solution. The reaction was started by the addition of H_2_O_2_. After incubation at 37°C for 1 h, the absorbance of solution was measured at 536 nm. Hydroxyl FRSA was evaluated as the inhibition rate of 1,10-phenanthroline-Fe^2+^ oxidation by hydroxyl radical. The FRSA was calculated using the following equation:


Where, A_sample_ is the absorbance in the presence of sample and H_2_O_2_; A_control_ is the absorbance in the presence of H_2_O_2_ without sample; A_blank_ is the absorbance without sample and H_2_O_2_. Each assay was performed in triplicate.

### Superoxide radical scavenging assay

The superoxide FRSA was evaluated by the method of Zhishen et al. (1999) with some variations. The system contained 2.4 ml of 62.5 mM sodium phosphate buffer (pH 7.8), 0.2 ml of 0.06 mM riboflavin, 0.1 ml of 0.003 mM ethylenediaminetetracetic acid disodium salt (EDTA), 0.2 ml of 1.125 mM nitroblue tetrazolium (NBT) and 0.05 ml of sample solution. The photo-induced reactions were performed in an aluminium foil-lined box with fluorescent lamps. The distance between the reactant and the lamp was adjusted until the intensity of illumination reached about 4000 lx. The reactant was illuminated at 25°C for 25 min. The photochemically-reduced riboflavin generated superoxide radical which reduced NBT to form blue formazan. Illuminated reaction mixture without a sample was used as a control. The reaction mixture was measured at 560 nm. The FRSA was calculated using the following equation:


Each assay was performed in triplicate.

### Determination of the content of polyphenolics

The polyphenolic contents were measured by the method of He and Zhang (1998). In a screw-capped tube, 4 ml of H_2_O and 5 ml of ferrous tartrate were added. Then 1 ml of aqueous extract and 15 ml of phosphate buffer (pH 7.5, 0.1 M) were added to give a total volume of 25 ml. The absorbance was measured at 540 nm. Results were expressed as mg catechin equivalents (CE) per gram dry weight. Each assay was performed in triplicate.

### Determination of the content of flavonoids

The spectrophotometer assay for the quantitative determination of flavonoid content was carried out as described by Zhishen et al. (1999). Briefly, the extract was diluted with 4 ml distilled water. At zero time, 0.3 ml 5% NaNO_2_ was added to the mixture. After 5 min, 3 ml 10% AlCl_3_ was added. After another 6 min, 2 ml 1 M NaOH was added and the total volume was made up to 10 ml with distilled water. Immediately, the solution was mixed well again and the absorbance of the mixture, pink in colour, was determined at 510 nm versus prepared water blank. Total flavonoids were expressed on a weight basis as mg catechin equivalents (CE) per gram dry weight. Each assay was performed in triplicate.

### Statistical analysis

Statistical analyses were performed according to the SPSS (version 11.5). Pearson’s correlation analysis was used to test for the significance of relationship. Values expressed were obtained from three independent experiments and averaged.

## Results

### Comparison of DPPH, hydroxyl, and superoxide radical scavenging activities

The scavenging activities of extracts from various flower materials on three free radicals, expressed as FRSA (%), were listed in Table 1.

Results showed that DPPH FRSA ranged from 8.119 ± 0.564% (*Panax notoginseng* (Burk.) F. H. Chen) to 94.244 ± 0.163% (*Rosa rugosa* Thunb), black tea was 85.322 ± 1.019%, and green tea was 93.191 ± 0.815%. *R. rugosa* Thunb, *Paeonia suffruticosa* Andr., *Paeonia lactiflora* Pall., *Trollius chinenses* Bunge*, Osmanthus fragrans* (Thunb.) Lour.*, Lonicera japonica* Thunb. showed higher FRSA (>50%) when compared with other extracts.

The scavenging activity of extracts on superoxide radical fluctuated between 0.405 ± 0.268% (*Jasminum sambac* (L.) Ait.) and 58.420 ± 0.842% (*O. fragrans* (Thunb.) Lour.), black tea was 48.139 ± 0.534%, and green tea was 59.169 ± 1.571%. *O. fragrans* (Thunb.) Lour. possessed the highest FRSA, followed by *R. rugosa* Thunb, *P. lactiflora* Pall., *P. suffruticosa* Andr. (>50%).

The reducing power of hydroxyl FRSA ranged from 33.513 ± 0.844% (*Chrysanthemum morifolium* Ramat) to 93.220 ± 0.346% (*Hibiscus sabdariffa* Linn.), black tea was 92.204 ± 0.253%, and green tea was 78.047 ± 1.847%. *H. sabdariffa* Linn. was found to have the highest FRSA, followed by *P. suffruticosa* Andr., *R. rugosa* Thunb., *P. lactiflora* Pall., *Lilium longiflorum* Thumb. (>80%).

### Polyphenolic and flavonoid contents

The total polyphenolic contents of the tested materials varied from 1.070 ± 0.148 mg CE/g (*L. longiflorum* Thumb.) to 96.208 ± 0.689 mg CE/g (*P. suffruticosa* Andr.) (Table 1), black tea was 60.704 ± 1.233 mg CE/g, and green tea was 88.356 ± 1.489 mg CE/g.

The total flavonoid contents of the tested materials varied from 10.837 ± 0.336 mg CE/g *C. morifolium* Ramat) to 83.797 ± 0.884 mg CE/g (*Trollius chinensis* Bunge) (Table 1), black tea was 60.288 ± 1.694 mg CE/g, and green tea was 26.667 ± 0.732 mg CE/g.

As observed from above results, 19 edible herbal flowers tested in this study exhibited antioxidant activities. *P. suffruticosa* Andr., *P. lactiflora* Pall., and *R. rugosa* Thunb. had obviously stronger FRSA activity and polyphenolic contents that were superior or comparable to black and green teas (Table 1). Our results were agreed with those observed by (Li et al. 2008, 2014) who also found those herbs had significant antioxidant properties and phenolic contents.

Polyphenolic content was found to be statistically significant with DPPH FRSA (*r* = 0.943, *P* < 0.01), superoxide FRSA (*r* = 0.833, *P* < 0.01), and hydroxyl FRSA (*r* = 0.500, *P* < 0.05). In addition, a significant relation was also detected between DPPH and superoxide FRSA (*r* = 0.897, *P* < 0.01), DPPH and hydroxyl FRSA (*r* = 0.555, *P* < 0.01), superoxide and hydroxyl FRSA (*r* = 0.486, *P* < 0.05) (Table 2). It suggested that phenolic compounds were largely responsible for total antioxidant capacity of the tested samples. The results were similar to previous reports that phenolic compounds were major antioxidant constituents in medicinal herbs, vegetables, fruits and spices (Cai et al. 2004; Huang et al. 2010; Li et al. 2013). However, there was no significant correlation between flavonoid contents and three tested free radicals (Table 2). Miliauskas et al. also reported that phenolic compounds were likely to contribute to the FRSA, and flavonoids showed only low correlation with FRSA and total amount of phenolics (Miliauskas et al. 2004).

**Table 2:** **Correlation analysis between polyphenolic content, flavonoid content and three free radicals**

		DPPH FRSA	Hydroxyl FRSA	Superoxide FRSA	Polyphenolic content	Flavonoid content
DPPH FRSA	Pearson Correlation	1				
	Sig. (2-tailed)	.				
Hydroxyl FRSA	Pearson Correlation	.555(**)	1			
	Sig. (2-tailed)	.009	.			
Superoxide FRSA	Pearson Correlation	.897(**)	.486(*)	1		
	Sig. (2-tailed)	.000	.026	.		
Polyphenolic content	Pearson Correlation	.943(**)	.500(*)	.837(**)	1	
	Sig. (2-tailed)	.000	.021	.000	.	
Flavonoid content	Pearson Correlation	.328	.217	.227	.194	1
	Sig. (2-tailed)	.147	.345	.322	.400	.

**Correlation is significant at the 0.01 level (2-tailed); *Correlation is significant at the 0.05 level (2-tailed).

Herbal flowers used in the test are often consumed in the form of teas. Herbal teas have been gaining popularity in western countries in recent years (Manteiga et al. 1997). Hundreds of different herbal teas are sold in health food stores. Available as pure or blended samples, herbal teas are popular because of their fragrance, antioxidant properties and therapeutic applications (Naithani et al. 2006). *Chrysanthemum indicum* L. has a long history for the treatment of inflammation, hypertension and respiratory diseases in China (Cheng et al. 2005). Flowers of *Chrysanthemum morifolium* Ramat are used as a Chinese natural medicine. Florists Chrysanthemum Flower (Ju Hua) is prescribed for anti-inflammatory, analgesic, and antipyretic purposes (Duh 1999). *Hibiscus sabdariffa* Linn. flowers are potentially a good source of antioxidant agents as anthocyanins (Ali et al. 2003). The roots of *Paeonia lactiflora* Pall. are commonly used in traditional Chinese medicine which showed to possess antispasmodic, anti-inflammatory and analgesic effects (Lee et al. 2005). Recent studies indicated that the extracts of *Paeonia lactiflora* Pall. flowers were also rich of polyphenols (Shu et al. 2014). The flowers of *Paeonia suffruticosa* Andr. are used in Chinese folk medicines for the treatment of diseases related mainly to irregular menstruation and dysmenorrhea (Huang 1994). Flowers and buds of *Lonicera japonica* Thumb., commonly known as Jinyinhua in traditional Chinese medicines, has been used for the treatment of affection by exopathogenic wind-heat or epidemic febrile disease at the early stage, sores, carbuncles, furuncles and swellings for centuries (Peng et al. 2005). *Osmanthus fragrans* (Oleaceae), also known as sweet olive, is a flower native to China, which is valued as an additive for tea and other beverages (Lee et al. 2007). Dried petals of *Rosa rugosa* Thunb. have been widely used as main material in preparation of rose teas in China, which was believed to provide nourishment and favor human health (Vinokur et al. 2006). Tea from *Camellia sinensis*, used as positive control in the test, is the most widely consumed beverage in the world, and it is an important dietary source of natural phenolic antioxidants (Lachman et al. 2003).

## Conclusion

In conclusion, The 19 edible flowers used in this study were carried as edible herbal tea resources and had been currently in commercial production in China. It was clearly demonstrated *Paeonia suffruticosa* Andr., *Paeonia lactiflora* Pall., and *Rosa rugosa* Thunb. had obviously stronger antioxidant activity and polyphenolic contents that were superior or comparable to black and green teas. Polyphenolic contents did correlate well with DPPH, superoxide, and hydroxyl FRSA. However, flavonoid contents did not correlate well with those FRSA. These findings can form the basis for further studies to isolate active compounds, and may contribute greatly to diversify and enhance the health-maintaining properties of the daily diet. However, in vivo studies are needed to confirm the health-promoting potential of these herbs.
